# Inhibition of iron overload-induced apoptosis and necrosis of bone marrow mesenchymal stem cells by melatonin

**DOI:** 10.18632/oncotarget.16382

**Published:** 2017-03-18

**Authors:** Fan Yang, Yuan Li, Gege Yan, Tianyi Liu, Chao Feng, Rui Gong, Ye Yuan, Fengzhi Ding, Lai Zhang, Elina Idiiatullina, Valentin Pavlov, Zhenbo Han, Wenya Ma, Qi Huang, Ying Yu, Zhengyi Bao, Xiuxiu Wang, Bingjie Hua, Zhimin Du, Benzhi Cai, Lei Yang

**Affiliations:** ^1^ Department of Orthopedics, The First Affiliated Hospital of Harbin Medical University, Harbin 150001, China; ^2^ Department of Pharmacology, College of Pharmacy, Harbin Medical University, Harbin 150081, China; ^3^ Department of Clinical Pharmacy, The Affiliated Second Hospital of Harbin Medical University, Harbin 150086, China; ^4^ Central Laboratory of Scientific Research, Bashkir State Medical University, Ufa 450008, Russia

**Keywords:** bone marrow mesenchymal stem cells, melatonin, iron overload, apoptosis, necrosis

## Abstract

Iron overload induces severe damage to several vital organs such as the liver, heart and bone, and thus contributes to the dysfunction of these organs. The aim of this study is to investigate whether iron overload causes the apoptosis and necrosis of bone marrow mesenchymal stem cells (BMSCs) and melatonin may prevent its toxicity. Perls’ Prussion blue staining showed that exposure to increased concentrations of ferric ammonium citrate (FAC) induced a gradual increase of intracellular iron level in BMSCs. Trypan blue staining demonstrated that FAC decreased the viability of BMSCs in a concentration-dependent manner. Notably, melatonin protected BMSCs against apoptosis and necrosis induced by FAC and it was vertified by Live/Dead, TUNEL and PI/Hoechst stainings. Furthermore, melatonin pretreatment suppressed FAC-induced reactive oxygen species accumulation. Western blot showed that exposure to FAC resulted in the decrease of anti-apoptotic protein Bcl-2 and the increase of pro-apoptotic protein Bax and Cleaved Caspase-3, and necrosis-related proteins RIP1 and RIP3, which were significantly inhibited by melatonin treatment. At last, melatonin receptor blocker luzindole failed to block the protection of BMSCs apoptosis and necrosis by melatonin. Taken together, melatonin protected BMSCs from iron overload induced apoptosis and necrosis by regulating Bcl-2, Bax, Cleaved Caspase-3, RIP1 and RIP3 pathways.

## INTRODUCTION

Bone marrow mesenchymal stem cells (BMSCs) are a type of stem cells which have a high ability to differentiate into a variety of cell types, including osteoblasts, chondrocytes and adipocytes [1]. A large body of evidence has showed that BMSCs transplantation offers a new therapeutic approach for a variety of diseases such as heart disease, cancer and bone defects [2–4]. Especially, the capability of BMSCs to transdifferentiate into osteoblasts makes it an ideal candidate in the reconstruction of bone defect [5]. It is also well documented that the dysfunction of BMSCs is closely associated with bone diseases such as osteoporosis and bone fracture. Various pathological factors such as oxidant stress, hyperhomocystemia and hyperglycemia can lead to the dysfunction and defects of BMSCs, and subsequently lead to the occurrence and development of bone diseases.

Iron is one of the most essential metals in human body, and it plays a crucial role in hemoglobin synthesis and some vital cellular processes including DNA and RNA formation, transportation of oxygen, carriage of nutrition and cellular environmental homeostasis [6]. The content of iron in human body is influenced by many factors such as frequent transfusions, iron supplement, organ dysfunction and chronic diseases. Iron deficiency will lead to the dysfunction of immune system, metabolic disorders, myasthenia and anemia [7–10]. By contrast, excess iron also damages several vital organs such as the liver, heart and bone [11–13]. Clinical studies have confirmed that iron overload is associated with numerous diseases such as hemochromatosis, liver injury, diabetes mellitus, cardiovascular disease and arthritis [14–18]. Recently, increasing evidence showed that iron overload is positively related with the incidence of osteoporosis [19]. The toxicity of iron overload in BMSCs has been shown to contribute to the progress of bone diseases. However, its underlying mechanisms and how to prevent its toxicity require further investigation.

Melatonin, mainly secreted by the pineal gland and released into blood, has been demonstrated to have strong antioxidant properties and many other strong protective effects on mammals [20]. Many studies have showed that melatonin plays an important role in many physiological processes such as regulating sleeping quality, heart rate, body temperature and immunity [21–24]. Besides, it is also reported that melatonin can regulate bone formation, mineralization and bone reconstruction [25–27]. However, the underlying mechanism that melatonin protects BMSCs against iron overload-induced dysfunction remains unclear. In this study, we firstly explored the molecular mechanism of iron overload-induced injury of BMSCs, and then investigated whether melatonin treatment protects BMSCs against the damage induced by iron overload.

## RESULTS

### Iron overload caused the decrease of cell viability in BMSCs

**Figure 1:**
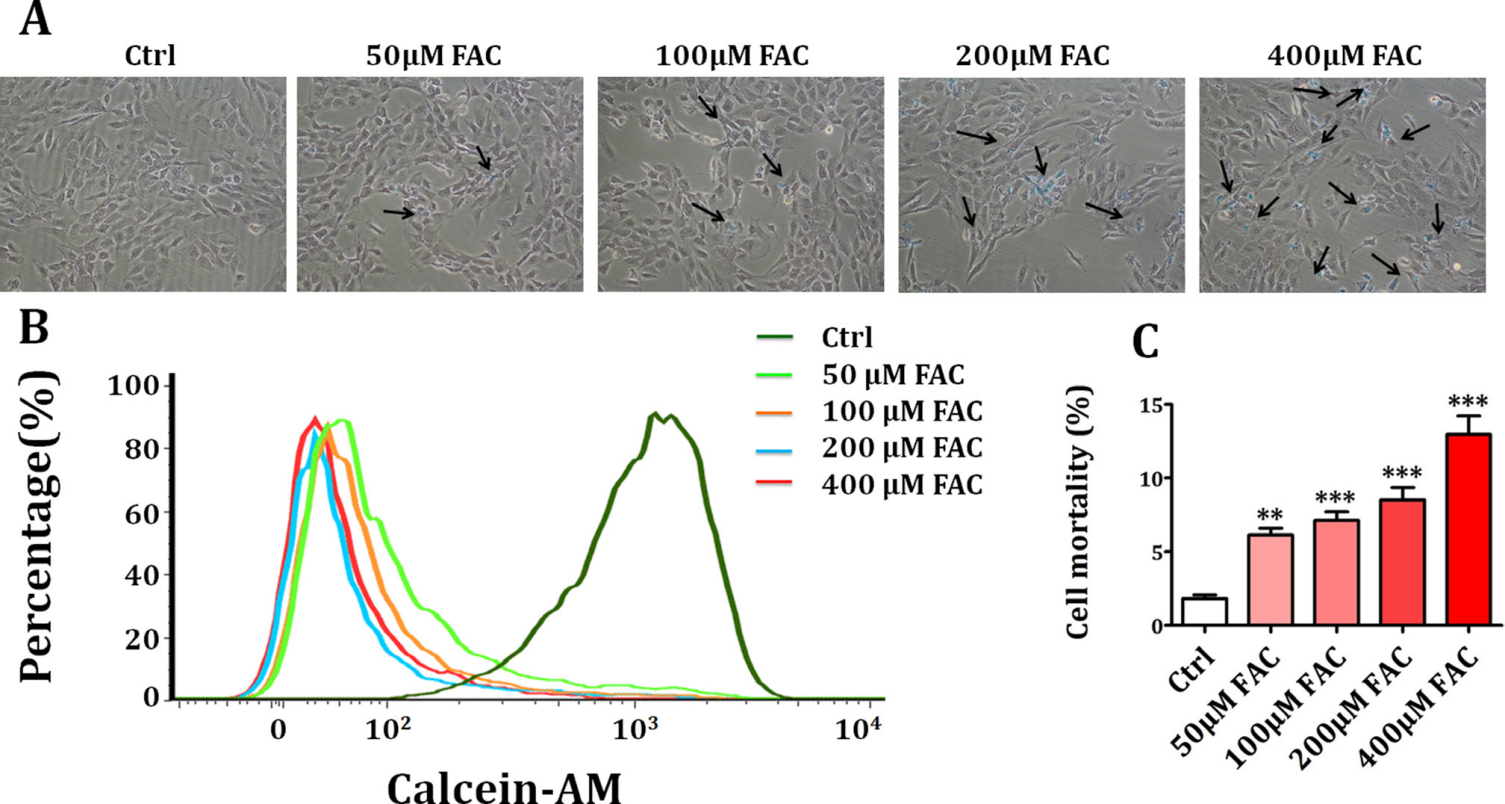
Iron overload caused the decrease of cell viability in BMSCs (**A**) Perls’ Prussion blue staining was applied to detect intracellular iron accumulation in FAC-induced BMSCs. After exposure to FAC 50 μM, 100 μM, 200 μM and 400 μM, intracellular iron level was gradually increased. (**B**) Flow cytometry analysis showed that treatment with FAC resulted in a concentration-dependent decrease of live BMSCs. (**C**) Trypan blue exclusion assay showed that exposure of BMSCs to FAC gradually decreased the number of live cells and increased the number of dead cells. Values are the mean ± SEM of 3 independent experiments (*n* = 3). **p < 0.01 versus Ctrl; ***p < 0.001 versus Ctrl.

Firstly, we investigated whether ferric ammonium citrate (FAC) could induce intracellular iron accumulation which was measured quantitatively by Perls’ Prussion blue staining. As shown in Figure 1A, after treatment with FAC 50 μM, 100 μM, 200 μM and 400 μM for 24 h, intracellular iron level of BMSCs was gradually increased. Then, we studied whether FAC-induced iron overload could decrease the viability of BMSCs. As shown in Figure 1B, flow cytometry analysis showed that FAC 50 μM, 100 μM, 200 μM and 400 μM treatment for 24 h resulted in a concentration-dependent decrease of live BMSCs. Meanwhile, trypan blue exclusion assay showed that exposure to FAC 50 μM, 100 μM, 200 μM and 400 μM for 24 h led to a gradual increase of dead BMSCs (Figure 1C). These results indicated that FAC-induced iron overload caused a concentration-dependent suppression on BMSCs viability.

### Protective effect of melatonin on FAC-induced cell death of BMSCs

**Figure 2:**
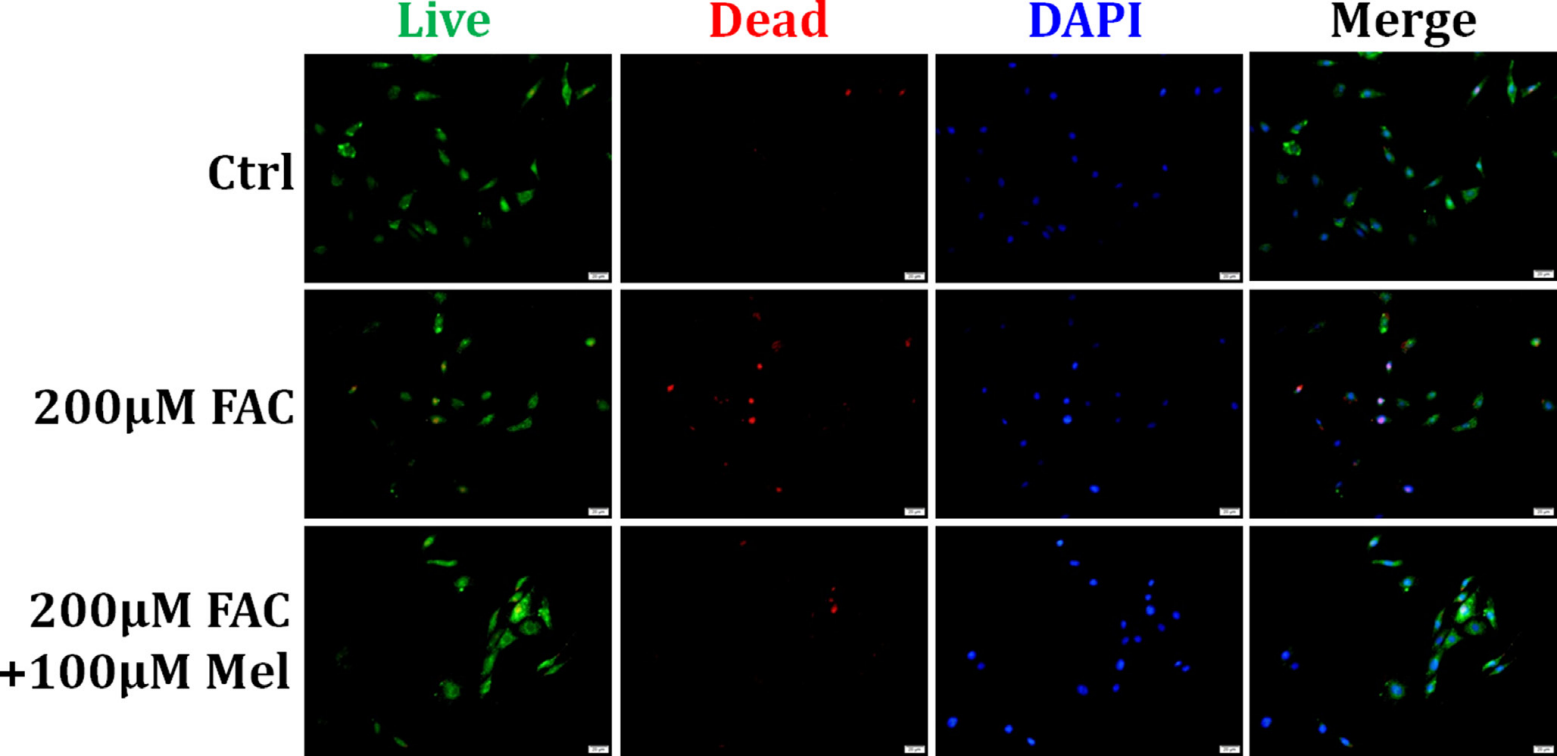
Protective effect of melatonin on FAC-induced cell death of BMSCs Live/Dead assay was applied to observe the effects of melatonin on FAC-induced cell death of BMSCs. Exposure of BMSCs to FAC 200 μM induced an increase of dead cells (red) compared to control group. But pretreatment with melatonin 100 μM abolished FAC-induced the increase of dead BMSCs. Data are for three independent experiments (*n* = 3).

Melatonin is a major chemical released by pineal gland, and has antioxidant, anti-apoptosis and anti-senescence activities [28]. We further investigated if melatonin treatment has protective effects on FAC-induced death of BMSCs by Live/Dead assay. In FAC 200 μM group, a significant increase of dead cells (red) was observed compared to the control group (Figure 2). However, pretreatment with melatonin 100 μM for 24 h significantly decreased the number of dead BMSCs in the presence of FAC (Figure 2). These results suggested that melatonin treatment exerts protective effects on FAC-induced cell death of BMSCs.

### Melatonin protected BMSCs against FAC-induced apoptosis and necrosis

**Figure 3:**
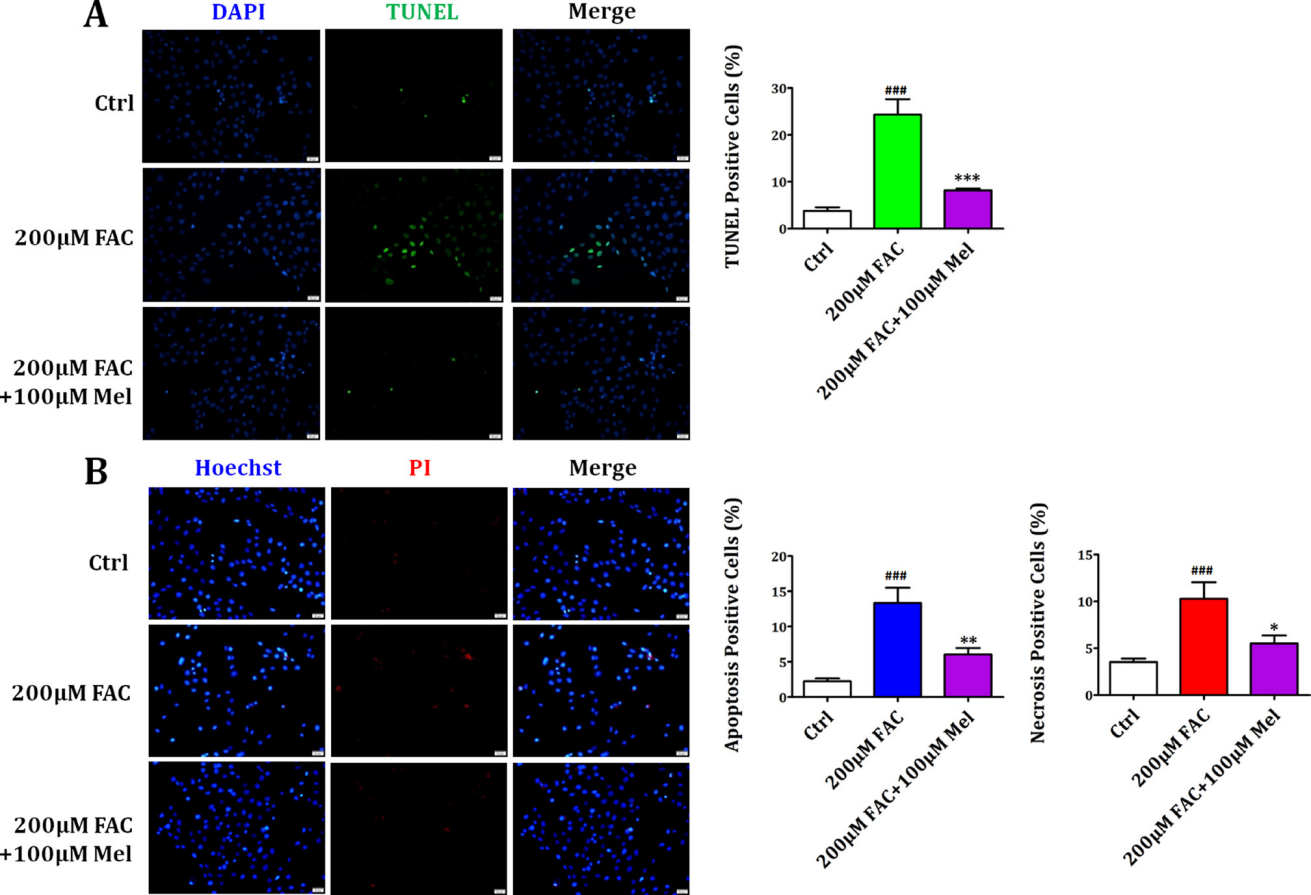
Melatonin protected BMSCs against FAC-induced apoptosis and necrosis (**A**) TUNEL staining showed that exposure of BMSCs to FAC 200 μM markedly increased the number of apoptotic positive cells compared to the control group. However, melatonin 100 μM led to a significant decrease in the number of apoptotic cells in FAC-induced BMSCs. (**B**) PI/Hoechst staining showed that exposure of BMSCs to FAC 200 μM for 24 h induced an increase of Hoechst-positive and PI-positive BMSCs. Afterwards, pretreatment with melatonin 100 μM countered the increase of apoptotic and necrotic positive cells induced by FAC 200 μM. Data are for three independent experiments (*n* = 3). ^###^*p* < 0.001, FAC 200 μM versus Ctrl; *p < 0.05, **p < 0.01, ***p < 0.001, FAC 200 μM + melatonin 100 μM versus FAC 200 μM.

We further investigated the contribution of apoptosis and necrosis to the protective effects of melatonin on FAC-induced reduced viability of BMSCs. TUNEL staining and Propidium Iodide (PI)/Hoechst staining were performed to detect the death form of BMSCs. As shown in Figure 3A, there were less TUNEL-positive (with green staining) cells in the control group. Whereas exposed to FAC 200 μM for 24 h, the number of TUNEL-positive cells was significantly increased. However, melatonin 100 μM treatment for 24 h suppressed the increase of TUNEL-positive cells with FAC treatment. PI/Hoechst staining also showed that after FAC 200 μM treatment for 24 h, the number of apoptotic BMSCs significantly increased (Figure 3B), and the number of necrotic BMSCs markedly increased as well. Nevertheless, after melatonin 100 μM treatment for 24 h, the increased apoptotic and necrotic-positive cells induced by FAC 200 μM were significantly reduced. These results suggested that melatonin could protect BMSCs against both apoptosis and necrosis induced by FAC.

### Melatonin protected BMSCs against FAC-induced intracellular ROS increase

**Figure 4:**
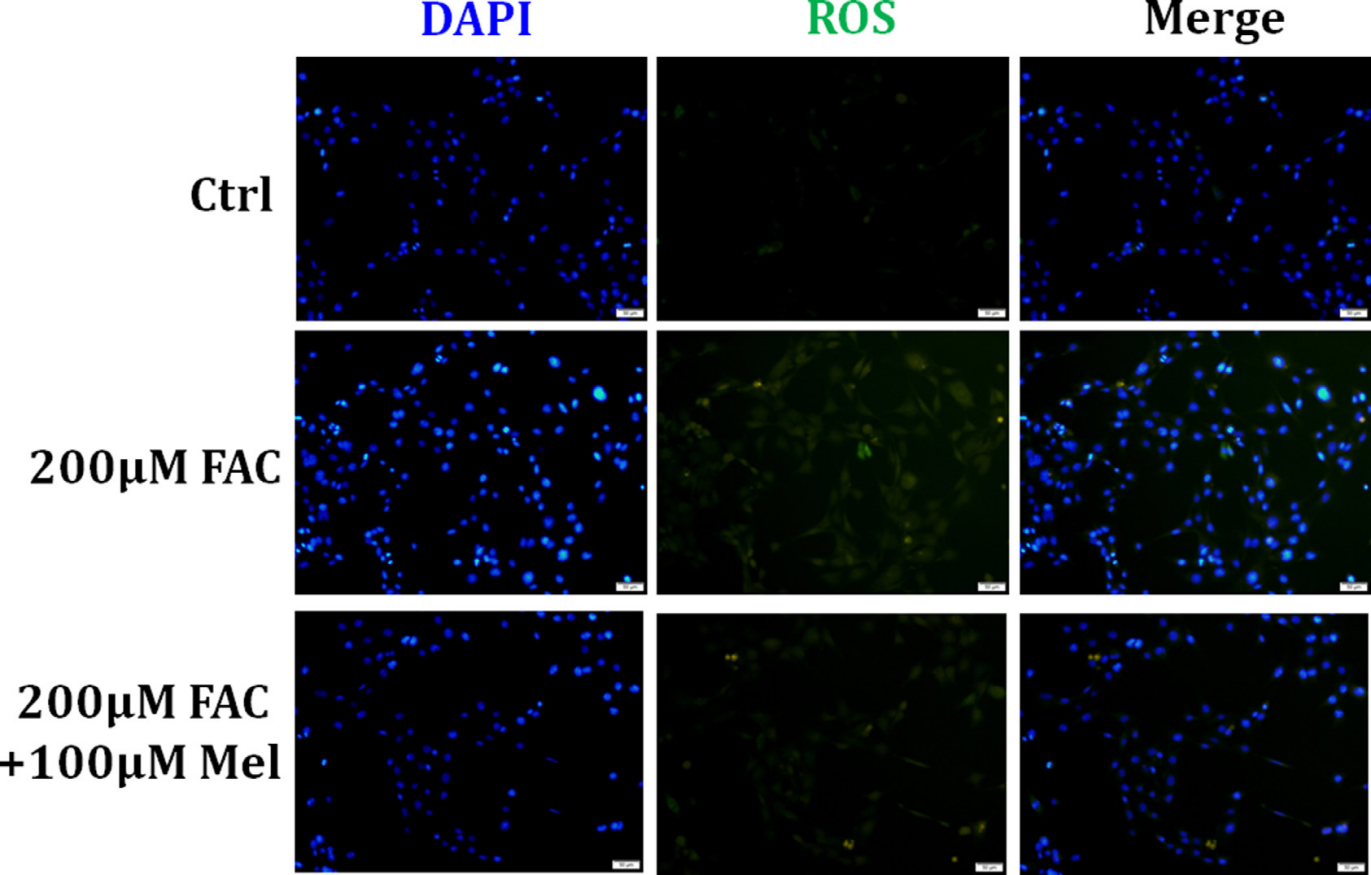
Melatonin protected BMSCs against FAC-induced intracellular ROS increase H2DCF-DA probe was applied to assessed the intracellular ROS production in FAC-induced BMSCs. ROS staining showed that exposed to FAC 200 μM for 24 h, BMSCs exhibited a gradual increase of intracellular ROS generation as illuminated by enhanced green staining in the cytoplasm. However, melatonin 100 μM suppressed the increase of ROS-positive cells with FAC treatment. Data are for three independent experiments (*n* = 3).

Growing evidence shows that excessive ROS production is responsible for cellular apoptosis and necrosis [29]. Thus, we assessed if intracellular ROS production of BMSCs was influenced by FAC and the protective effect of melatonin. As displayed in Figure 4, after exposing to FAC 200 μM for 24 h, BMSCs exhibited a gradual increase of intracellular ROS generation as illuminated by enhanced green staining in the cytoplasm. However, melatonin 100 μM treatment for 24 h suppressed the increase of ROS-positive cells with FAC treatment. These results suggested that melatonin pretreatment in FAC-induced BMSCs significantly prevented the rise in ROS production, in parallel with the decreased of apoptosis and necrosis induced by FAC.

### Melatonin suppressed FAC-induced BMSCs death by regulating Bcl-2/Bax, Cleaved Caspase-3 and RIP pathways

**Figure 5:**
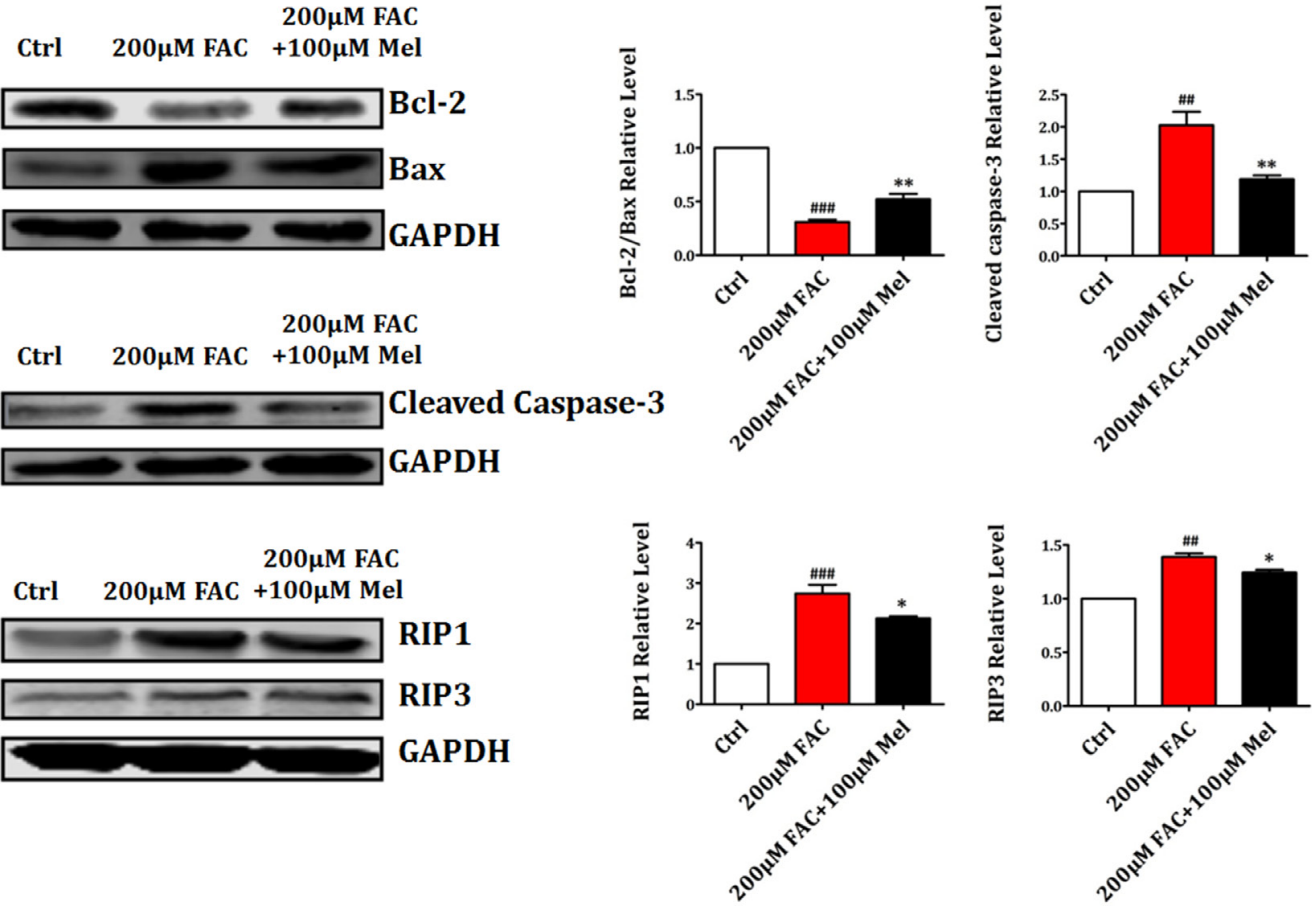
Melatonin suppressed FAC-induced BMSCs death by regulating Bcl-2/Bax and RIP pathways Western blot showed that the exposure of BMSCs to FAC 200 μM caused a significant downregulation of Bcl-2 and upregulation of Bax, Cleaved Caspase-3, RIP1 and RIP3 proteins. However, pretreatment with melatonin 100 μM suppressed the decreased expression of Bcl-2, and the increased expression of Bax, Cleaved Caspase-3, RIP1 and RIP3 in FAC-treated BMSCs. Values are the mean ± SEM of 3 independent experiments (*n* = 3). ^##^*p* < 0.01, ^###^*p* < 0.001, FAC 200 μM versus Ctrl, *p < 0.05, **p < 0.01, FAC 200 μM + melatonin 100 μM versus FAC 200 μM.

Then we further employed western blot to study the expression levels of apoptosis-related proteins such as Bcl-2, Bax and Cleaved Caspase-3 and necrosis-related proteins such as RIP1 and RIP3. As shown in Figure 5, there was a significant downregulation of Bcl-2 and upregulation of Bax and Cleaved Caspase-3 in FAC 200 μM group compared to control group. However, melatonin pretreatment for 24 h suppressed the reduction of Bcl-2 and the increase of Bax and Cleaved Caspase-3 in FAC-treated BMSCs. In addition, the expression of necrosis-related proteins RIP1 and RIP3 was significantly increased by FAC 200 μM treatment for 24 h. However, the increased expressions of RIP1 and RIP3 were was also attenuated by melatonin 100 μM. These results indicated that melatonin could suppress the activation of Bax, Cleaved Caspase-3 and RIP pathways in FAC-treated BMSCs.

### Effects of melatonin inhibitor luzindole on FAC-induced BMSCs death

**Figure 6:**
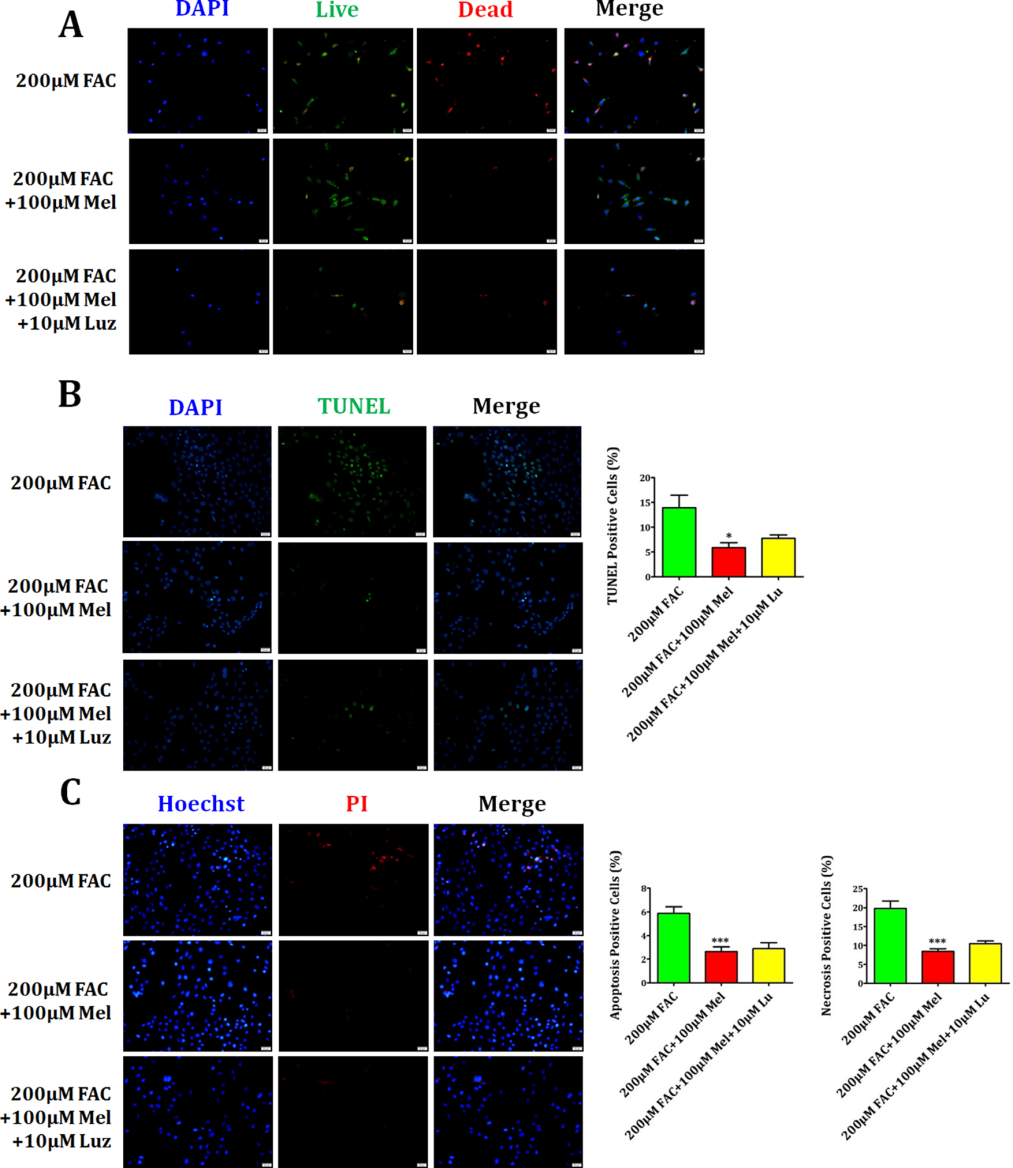
Effects of melatonin inhibitor luzindole on FAC-induced BMSCs death (**A**) Live/Dead staining showed that melatonin 100 μM induced the decrease of dead cells with red staining, which can not be abrogated by the melatonin receptor blocker luzindole 10 μM. (**B**) TUNEL staining confirmed that FAC 200 μM led to significant increase in the number of TUNEL positive cells, which could be obviously reversed by melatonin 100 μM. However, luzindole failed to suppress the protective function of melatonin in FAC-treated BMSCs. (**C**) PI/Hoechst staining showed that luzindole 10 μM failed to block the protection of melatonin 100 μM on FAC-induced apoptosis and necrosis of BMSCs. Data are for three independent experiments (*n* = 3). *p < 0.05, ***p < 0.001, FAC 200 μM + melatonin 100 μM versus FAC 200 μM.

Previous studies have reported that melatonin produces biological activities in stem cells in a melatonin receptor-dependent or independent manner [30]. In this study, we further studied if melatonin receptor is involved in the protection of FAC-induced BMSCs apoptosis and necrosis by melatonin. As shown in Figure 6A, Live/Dead staining showed a significant decrease of dead cells with red staining in melatonin 100 μM group compared to FAC 200 μM group. But melatonin receptor blocker luzindole 10 μM treatment for 24 h did not block the protective effects of melatonin on FAC-treated BMSCs. Consistently, TUNEL staining also confirmed that FAC 200 μM led to a significant increase in the number of TUNEL positive cells, which was obviously reversed by melatonin 100 μM. However, luzindole failed to abolish the protective effects of melatonin on FAC-treated BMSCs (Figure 6B). Likewise, Figure 6C demonstrated that protective effects of melatonin on FAC-induced the increase of apoptotic Hoechst-positive BMSCs and necrotic PI-positive BMSCs was not blocked by luzindole 10 μM treatment for 24 h. Therefore, these results indicated that melatonin produces the protective effect on FAC-induced BMSCs death in a melatonin receptor-independent manner.

### Luzindole has no effects on apoptotic and necrotic proteins in BMSCs

**Figure 7:**
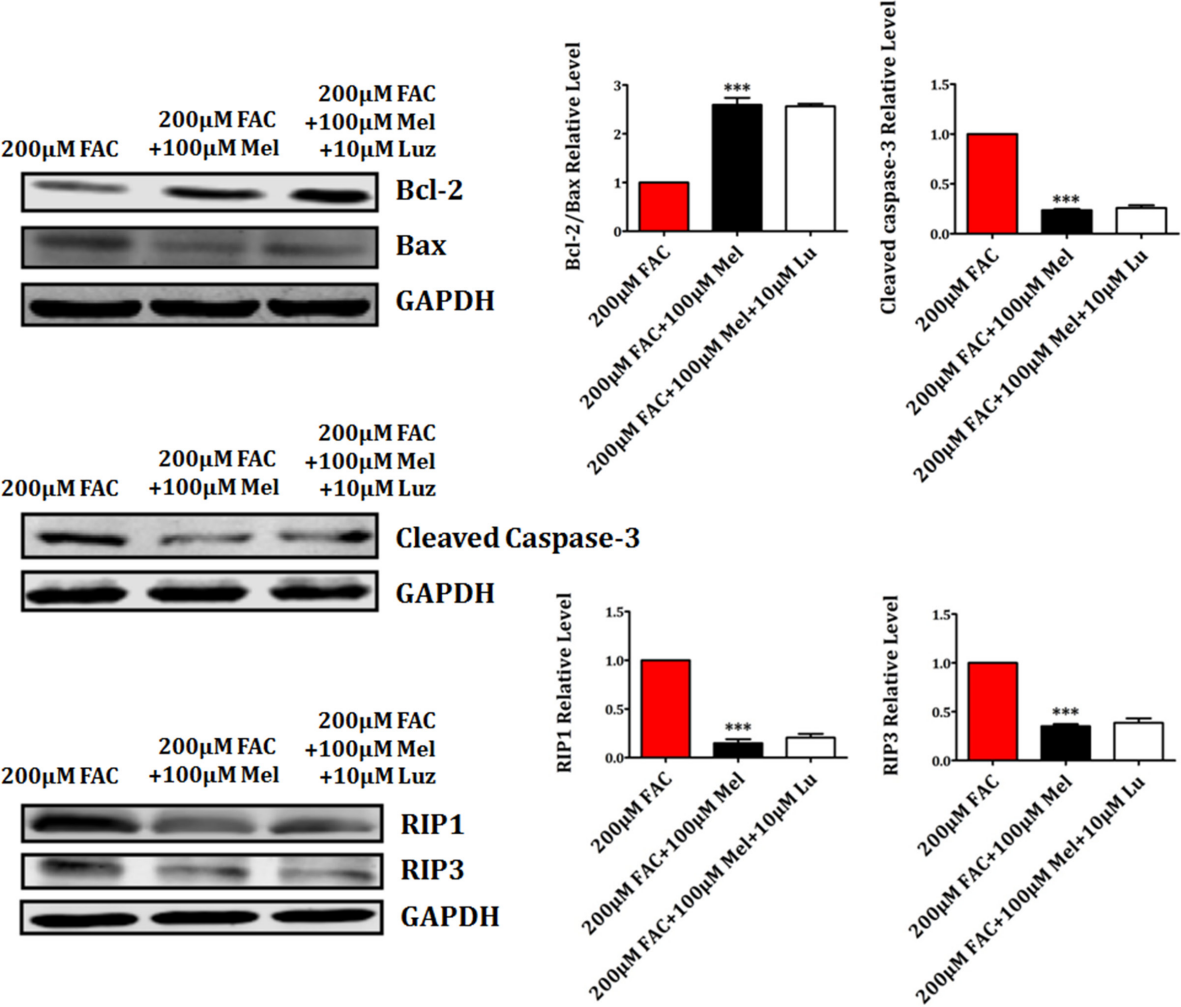
Luzindole has no effects on apoptotic and necrotic proteins in BMSCs Western blot showed that the expression of Bax, Cleaved Caspase-3, RIP1 and RIP3 were significantly decreased by melatonin 100 μM. The increased expression of Bcl-2, decreased expression of Bax, Cleaved Caspase-3, RIP1 and RIP3 in melatonin pretreatment were not reversed by luzindole 10 μM. Values are the mean ± SEM of 3 independent experiments (*n* = 3). ***p < 0.001, FAC 200 μM + melatonin 100 μM versus FAC 200 μM.

The influence of luzindole on the expression of apoptosis-related proteins Bcl-2, Bax and Cleaved Caspase-3, and necrosis-related proteins RIP1 and RIP3 was further studied. We found that melatonin 100 μM treatment for 24 h induced the upregulation of anti-apoptotic protein Bcl-2 and the downregulation of pro-apoptotic protein Bax and Cleaved Caspase-3 (Figure 7). However, the increase of Bcl-2 and the decrease of Bax and Cleaved Caspase-3 were not attenuated by luzindole 10 μM treatment for 24 h. In addition, the expression of necrotsis-related RIP1 and RIP3 was significantly decreased by melatonin 100 μM 24 h treatment. Likewise, luzindole failed to block melatonin-induced the reduction of RIP1 and RIP3 in FAC-treated BMSCs (Figure 7). These results sugggested that melatonin protected BMSCs against apoptosis and necrosis induced by FAC in a melatonin receptor-independent manner.

## DISCUSSION

The present study firstly revealed that melatonin is able to ameliorate FAC-induced apoptosis and necrosis of BMSCs by decreasing the intracelluar ROS level and regulating apoptotic and necrotic proteins. These findings provide a better understanding of the mechanisms and therapeutics of iron overload-induced BMSCs apoptosis and necrosis.

Iron is required for the development and physiology of human body [31]. It has been well recognized that iron is a double-edged sword for human health. For example, iron deficiency leads to the dysfunction of immune system, metabolic disorders, myasthenia and anemia [32]. On the contrary, excessive iron damages several vital organs such as liver, heart and bone [11–13]. In clinical, a number of studies have shown that iron overload is associated with bone diseases such as osteoporosis [33]. However, the underlying mechanism why iron overload contributes to the incidence of osteoporosis still remains unclear.

Bone marrow mesenchymal stem cells (BMSCs) as one kind of adult stem cells are capable of differentiating into a variety of cells such as cardiomyocytes, osteoblasts, chondrocytes and adipocytes. Recent studies have showed that BMSCs played a supporting role in bone development and formation [34]. The dysfunction of BMSCs may lead to the imbalance of bone metabolism and subsequent induce bone disorders [35]. Therefore, in the present study, we hypothesized that FAC-induced iron overload might contribute to the apoptosis and necrosis of BMSCs.

Firstly, we found that exposure to FAC 50 μM, 100 μM, 200 μM and 400 μM induced a gradual increase of intracellular iron level in BMSCs. Then flow cytometry analysis and trypan blue exclusion assay were performed to confirm whether FAC-induced iron overload could decrease the viability of BMSCs. The results demonstrated that FAC caused a concentration-dependent reduction of BMSCs viability, accompanied with intracellular iron accumulation. Melatonin is mainly secreted by the pineal gland, and have many physiological functions such as regulating sleeping quality, heart rate, body temperature and immunity [36]. Besides, it has been reported that melatonin can regulate bone formation, mineralization and bone reconstruction [37–39]. Whether melatonin treatment can protect BMSCs against the damage induced by iron overload remained unknown. Therefore, we further observed the effect of melatonin on FAC-induced BMSCs death. We found that the number of dead BMSCs in the presence of FAC 200 μM was significantly decreased by pretreatment with melatonin 100 μM. It indicates that melatonin treatment exerted resistant and protective effects on FAC-induced death of BMSCs. To our knowledge, this is the first study that uncovers the protective effects of melatonin on FAC-induced BMSCs death. Then, exposure to FAC 200 μM significantly increased the number of TUNEL-positive cells. However, melatonin 100 μM led to a significant decrease of apoptotic cells in FAC-induced BMSCs. It suggests that melatonin inhibits the apoptosis of BMSCs caused by iron overload. Furthermore, we found that after FAC 200 μM treatment for 24 h, the number of Hoechst-positive and PI-positive BMSCs was markedly increased. But, melatonin 100 μM significantly suppressed the increased apoptotic and necrotic positive cells induced by FAC 200 μM. These results indicates that melatonin protects BMSCs against both apoptosis and necrosis induced by FAC. Likewise, melatonin has been shown to protect endothelial progenitor cells against tumor growth factor-β-induced apoptosis and necrosis [40].

Numerous reports have demonstrated that iron overload led to an increase in cellular ROS and negatively affected vital organs, such as the liver, heart and bone. Keeping ROS at an appropriate level plays an important role in some biological phenomena, such as the activation of signaling pathways. ROS could trigger a variety of signal transduction pathways, such as MAPK and p53/p21, and eventually leading to cell apoptosis. Similarly, it was previously reported that FAC treatment may induce the increase of intracelluar ROS in hematopoietic stem cells and progenitor cells [41]. Then, we further found that the increased intracelluar ROS levels of BMSCs induced by FAC 200 μM was partially attenuated by melatonin 100 μM, indicating that ROS are involved in the apoptosis and necrosis of BMSCs by FAC-induced iron overload. In agreement, it was previously reported that iron overload caused apoptosis of umbilical cord-derived mesenchymal stem cells [42]. But, this is the first time to confirm that iron overload also induces necrosis of mesenchymal stem cells.

In addition, previous studies have showed that the downregulation of anti-apoptotic protein Bcl-2 and the upregulation of pro-apoptotic protein Bax and Cleaved Caspase-3 are critical for cellular apoptosis. Thus, we further employed western blot to detect the expression of these proteins in the presence and absence of melatonin. The results showed that FAC treatment induced a significant downregulation of Bcl-2 and upregulation of Bax and Cleaved Caspase-3 in BMSCs, but melatonin reversed this alteration. The expression of necrosis-related proteins RIP1 and RIP3 was also significantly increased by FAC 200 μM. However, melatonin suppressed the increased expression of RIP1 and RIP3. These results confirmed that melatonin could protect BMSCs against apoptosis and necrosis induced by FAC through modulating apoptotic and necrotic proteins.

Then, Live/Dead, TUNEL and PI/Hoechst stainings were performed to better understand whether the protective effects of melatonin in FAC-induced apoptosis and necrosis of BMSCs depend on melatonin membrane receptor. Live/Dead assay showed that the number of dead cells was obviously inhibited by melatonin 100 μM, which can not be altered by the melatonin receptor blocker luzindole 10 μM. TUNEL and PI/Hoechst stainings also confirmed that luzindole 10 μM failed to abrogate the protective effect of melatonin on FAC-induced apoptosis and necrosis of BMSCs. Western blot also showed that melatonin-induced the upregulation of Bcl-2 and the downregulation of Bax and Cleaved Caspase-3, which were not altered by the melatonin receptor blocker luzindole 10 μM. Similarly, the reduction of RIP1 and RIP3 proteins by melatonin 100 μM were not obviously abolished by luzindole 10 μM. These results suggest that melatonin protects BMSCs against apoptosis and necrosis induced by FAC in a melatonin receptor-independent manner.

## MATERIALS AND METHODS

### Animals

Male C57BL/6J (18–20 g) mice were purchased from the experimental animal center of Harbin Medical University. All animals protocol was approved by the Guide for the Care and Use of Laboratory Animals published by the US National Institute of Health. Additionally, all experimental procedures were carried out in strict in accordance with the ethic committee of Harbin Medical University.

### Reagents

Melatonin, ferric ammonium citrate (FAC) and luzindole were purchased from Sigma (St, Louis. Mo. USA). Melatonin, FAC and luzindole were dissolved in dimethyl sulfoxide (DMSO) and the final culture concentration of DMSO was ≤ 0.1%. Other chemicals were all purchased from Sigma (St, Louis. Mo. USA). Stem cell medium was purchased from Stem Cell (Canada). Calcein-AM (40719ES60) was purchased from Shanghai YEASEN Biotechnology. Trypan blue (T-6146) was purchased from Sigma (St, Louis. Mo. USA). Live/Dead Kit (1736918) was purchased from Life technologies. Terminal Deoxynucleotidyl Transferase-Mediated dUTP Nick-End Labeling (TUNEL) was purchased from Roche Company (Roche, Germany). PI/Hoechst staining (CA1120) was purchased from Solarbio (Beijing Solarbio Science & Technology, China).

### Isolation and culture of BMSCs

BMSCs were primarily isolated and cultured from Male C57BL/6J mice as previously described [43]. Briefly, BMSCs were harvested by flushing the femurs and tibias with BMSCs culture medium. Then, the culture medium was collected, eliminating the thrombus, seeded into 25 cm^2^ flasks, and cultured at 37°C in a humidified atmosphere with 95% air and 5% CO_2_. The culture medium was replaced every 3 days, during which non-adherent cells were discarded. After 7–10 days culture, when the cells reached 70% confluency, BMSCs were trypsinized and passaged. All experiments were performed using cultured BMSCs from third to fifth passage.

### Perls’ Prussion blue staining

Intracellular iron accumulation was measured by Perls’ Prussion blue staining. The cells were treated with dilute hydrochloric acid to release ferric ion from binding proteins. Then these ions reacted with potassium ferrocyanide to produce an insoluble blue compound. Briefly, the BMSCs were washed by PBS for 3 times, and then fixed in 4% paraformaldehyde solution for 30 min at room temperature. Next, the cells were incubated with the Perls’ Prussion blue solution for 30 min in dark. After washed in PBS for 3 times, the BMSCs were incubated with eosin staining solution for 30 s. Bright light images were taken under microscope.

### Flow cytometry analysis

The fluorescence intensity associated with iron overload was quantified using Calcein-AM according to the manufacturer's instructions. Briefly, cells were digested when the confluency reached 90%, and centrifuged at 1000 rpm for 5 min. Then the supernatant was discarded and the sendiment was washed by cold PBS for 3 times. Next, the precipitant of each tubes was incubated by appropriate Calcein-AM, which was diluted 1:1000 from manufacture's stock, at 37°C in dark for 30 min. Then cells were washed and resuspended in PBS. After that, BMSCs were immediately analyzed using a Cytoflex flow cytometer (Becton-Dickinson, Sunnyvale, CA, USA). The negative control alone was analyzed prior to sample acquisition to ensure that all background noise was eliminated from the cells.

### Trypan blue assay

The cell death was evaluated using trypan blue assay. Trypan blue is a commonly used biological staining reagent, which can detect the membrane integrity of cells. Under normal condition, the live cells exclude the trypan blue, while the dead cells were stained with trypan blue because of the complete loss of membrane permeability. Trypan blue assay was applied according to the manufacturer's instructions. Briefly, BMSCs were plated in the 6-well plates (5 × 10^5^ cells per well) and incubated for 24 h. After being treated, the cells were detached with 300 μL trypsin-EDTA solution. The mixture of detached cells were washed by PBS for 3 times and centrifuged at 1000 rpm for 5 min. Then the residue was combined with 800 μL 0.4% trypan blue solution (pH 7.2–7.3) and dispersed. After 3 min staining, cells were counted using an automated cell counter (TC10, BioRad). The dead cells were stained with blue color. Cell mortality (%) was expressed as percentage of the dead cell number/the total cell number.

### Live/Dead staining

Live/Dead staining was performed to evaluate the viability in FAC-treated BMSCs. According to the manufacture's protocol, the cells were stained with the live/dead reagent, which labeled live cells and dead cells respectively. Then the cells were incubated at room temperature in dark for 30 min prior to analysis. Cell were then quickly washed by PBS following incubation, and examined under a fluorescence microscope. The live cells were labeled by Calcein-AM with green fluorescence and the dead cells were labbled by ethidium homodimer with red fluorescence. For each sample, ten randomly selected areas were counted and the average value was calculated.

### Terminal Deoxynucleotidyl Transferase-Mediated dUTP Nick-End Labeling (TUNEL) assay

TUNEL assay was performed according to the manufacturer's instructions (Roche, Germany). Briefly, BMSCs were fixed with 4% paraformaldehyde solution for 15 min at room temperature, then permeabilized in 0.1% Triton X-100. Next, cells were stained by freshly prepared TUNEL reaction mixture for 1 h at 37°C in a humidified atmosphere and protected from light. Then the cells were incubated in DAPI for 20 min to stain nuclei. Finally, cells were immediately analyzed under a fluorescence microscope to view the green fluorescence of apoptotic cells at 520 nm and blue DAPI stained nuclei at 460 nm. For each sample, ten randomly selected areas were counted and the average value was calculated.

### Propidium Iodide (PI)/Hoechst 33342 staining

The PI/Hoechst staining assay was performed using PI/Hoechst staining (Beijing Solarbio Science & Technology, China) according to the manufacturer's instructions. Briefly, 10 μM PI was added to BMSCs for 2 h. Cells were then washed with PBS by 3 times, followed by 4% paraformaldchyde fixation. Then, BMSCs were incubated with 2 mg/mL glycine, washed with PBS twice. After permeabilization with PBS containing 0.5% Triton X-100 and extensive washed by PBS, cells were incubated with staining solution for 30 min. Next, cells were washed with PBS containing 0.5% Triton X-100 for 3 times, followed by 10 min incubation with Hoechst 33342. Images of the staining were captured with a fluorescent microscope (Olympus DP71X). For each sample, ten randomly selected areas were counted and the average value was calculated.

### Measurement of Reactive Oxygen Species (ROS)

To quantify the intracellular production of ROS in FAC-induced BMSCs, we applied 2,7-dichlorodihydrofluorescein diacetate (H2DCF-DA) probe, a fluorogenic dye which was used for the measurement of ROS within the cells. The method to measure ROS generation was just as previously described [44]. Briefly, after rinsing 3 times with PBS, the cells were loaded with H2DCF-DA 10 μM for 30 min at 37°C in dark. After fixed in 4% paraformaldehyde, and washing with PBS for 3 times, the cells were incubated in DAPI for 20 min to stain nuclei. Finally, cells were analyzed under a fluorescence microscope (Eclipse TE300, Nikon, Japan). For each sample, ten randomly selected areas were counted and the average value was calculated.

### Western blot

The protocol for western blot was based on previous reports. Briefly, the BMSCs were lysed in ice-cold cell lysis buffer containing with protease inhibitors, and the protein concentration in cell extracts was quantified using a BCA protein assay kit following the manufacturer's recommended protocol. Equal amounts (70 μg) of protein from each extract were denatured and resolved using a 12.5% SDS-PAGE, and then transferred by electrophoresis onto a nitrocellulose (NC) membrane (Millipore, Billerica, MA). Nonspecific proteins were blocked by incubating the membrane in blocking buffer (5% nonfat dry milk in T-TBS containing 0.05% Tween-20) for 1 h at room temperature and then the NC membranes were incubated overnight at 4°C with primary antibodies against: Bcl-2 (1: 1000, Abcam), Bax (1: 1000, Abcam), Cleaved Caspase-3 (1: 1000, Abcam), RIP1 (1:1000, Abcam), RIP3 (1:1000, Abcam) and GAPDH (1:1000, Abcam). After rinsing, the membranes were incubated in Horseradish peroxidase-conjugated secondary antibodies (1:2000, Cell Signaling Technology) as a secondary antibody for 1 h incubation at room temperature. The washing procedure was repeated 6 times within one hour. Immunoreactive bands were visualized by enhanced chemiluminescence (ECL; Amersham Biosciences). For purposes of quantification, The intensity of bands was quantified using the ImageJ Software (National Institutes of Health, Bethesda, MD, USA).

### Statistical analysis

Data were analyzed using GraphPad Prism-5 software. All of the experimental data were expressed as mean ± SEM, and each experiment was performed from 3 to 10 independent groups, number of samples (n) for each experiment was indicated in the corresponding figure legend. One-way ANOVA was used to determine statistical significance, ^#^*p* < 0.05, ^##^*p* < 0.01, ^###^*p* < 0.001, *p < 0.05, **p < 0.01 and ***p < 0.001 were considered to be statistically significant.

## CONCLUSIONS

In the present study, we uncovered that iron overload induced by FAC led to the apoptosis and necrosis of BMSCs, which was effectively rescued by melatonin. This study helps us to better understand the mechanisms underlying FAC-induced BMSCs death, and suggests new application of melatonin in clinics.

## Abbreviations

BMSCs: Bone marrow mesenchymal stem cells
FAC: ferric ammonium citrate
ROS: reactive oxygen species
PI: propidium iodide
DMSO: dimethyl sulfoxide
TUNEL: Terminal Deoxynucleotidyl Transferase-Mediated dUTP Nick-End Labeling
NC: nitrocellulose
